# Tetrahydrobiopterin modulates ubiquitin conjugation to UBC13/UBE2N and proteasome activity by S-nitrosation

**DOI:** 10.1038/s41598-018-32481-4

**Published:** 2018-09-25

**Authors:** Jade Bailey, Simon Davis, Andrew Shaw, Marina Diotallevi, Roman Fischer, Matthew A. Benson, Hanneng Zhu, James Brown, Shoumo Bhattacharya, Benedikt M. Kessler, Keith M. Channon, Mark J. Crabtree

**Affiliations:** 1 BHF Centre of Research Excellence, Division of Cardiovascular Medicine, Radcliffe Department of Medicine, John Radcliffe Hospital, University of Oxford, Oxford, OX3 9DU UK; 2 Target Discovery Institute, Nuffield Department of Medicine, University of Oxford, Roosevelt Drive, Oxford, OX3 7FZ UK

## Abstract

Nitric Oxide (NO) is an intracellular signalling mediator, which affects many biological processes via the posttranslational modification of proteins through S-nitrosation. The availability of NO and NOS-derived reactive oxygen species (ROS) from enzymatic uncoupling are determined by the NO synthase cofactor Tetrahydrobiopterin (BH4). Here, using a global proteomics “biotin-switch” approach, we identified components of the ubiquitin-proteasome system to be altered via BH4-dependent NO signalling by protein S-nitrosation. We show S-nitrosation of ubiquitin conjugating E2 enzymes, in particular the catalytic residue C87 of UBC13/UBE2N, leading to impaired polyubiquitylation by interfering with the formation of UBC13~Ub thioester intermediates. In addition, proteasome cleavage activity in cells also seems to be altered by S-nitrosation, correlating with the modification of cysteine residues within the 19S regulatory particle and catalytic subunits of the 20S complex. Our results highlight the widespread impact of BH4 on downstream cellular signalling as evidenced by the effect of a perturbed BH4-dependent NO-Redox balance on critical processes within the ubiquitin-proteasome system (UPS). These studies thereby uncover a novel aspect of NO associated modulation of cellular homeostasis.

## Introduction

Nitric Oxide (NO), produced by endothelial nitric oxide synthase (eNOS), is a key mediator of vascular homeostasis, and NO bioavailability is reduced early in vascular disease states such as hypercholesterolemia, diabetes and hypertension, as well as throughout the progression of atherosclerosis^1–3^. This is a result of both decreased NO synthesis and increased NO consumption by reactive oxygen species (ROS) such as superoxide. In these disease states, eNOS may become enzymatically ‘uncoupled’ and generates superoxide rather than NO, contributing to vascular oxidative stress and endothelial dysfunction^4,5^. This switch between NO and ROS production, and the subsequent perturbation of NO-Redox balance, is determined by the availability of the essential cofactor tetrahydrobiopterin (BH4)^6,7^. As these redox sensor and effector functions of BH4 expand the repertoire of NOS signalling to include not only NO, but also ROS, we therefore propose NOS/BH4 as a redox ‘hub’ linking upstream redox-sensitive effects of BH4 with redox-dependent targets and pathways that lie downstream^8^.

NO and ROS signal through chemical reactions with specific atoms of target proteins that lead to modification of metal centres and to covalent protein modifications. This notion of NO–Redox balance may be defined by the idea that reactive nitrogen species (RNS) and ROS work together in biological systems to achieve optimal signalling. NO-Redox imbalance arises when cellular signalling is disrupted by perturbed ROS/RNS. Moreover, cross-talk exists between the enzymes that produce ROS and RNS, so NO deficiency can in some cases result in increased ROS production. Thus, the interactions between ROS and RNS are multifaceted and strike a balance that can be disrupted at both the cell and organ levels in cardiovascular disease states, as within the dysfunctional endothelium. A key concept in understanding redox balance in the cardiovascular system is that the effects of ROS and RNS depend on the location, amount, and timing of their production.

NOS is the major source of NO in mammalian cells, and activation of the enzyme following stimulation mediates a broad range of signalling pathways. In addition to the classical interaction with haem proteins such as sGC, NO exerts physiological actions through the posttranslational modification and regulation of proteins, and a large body of evidence now demonstrates that s-nitrosation of Cys residues within a wide spectrum of proteins determines the ubiquitous influence of NO on cellular function^9,10^. For instance, the NO-haem interaction activates soluble guanylate cyclase (sGC), and in this way stimulates the synthesis of GMP, the subsequent activation of protein kinase G and the modulation of phosphorylation-dependent signalling^11,12^. Furthermore, activation of the transcription factor NF-E2-related factor (NRF2) is achieved by inactivation of its cognate ubiquitin E3 ligase complex, triggered by S-nitrosation of critical cysteine residues in the Kelch-like ECH-associated protein (Keap1), leading to the disassembly of its association with the Cullin 3 ubiquitin E3 ligase complex^13–15^. Stabilised NRF2 levels subsequently lead to the induction and activation of antioxidant defence genes. Incomplete understanding of the balance among NO, ROS, and downstream redox effects may underlie the inconsistent effects of “antioxidant” therapies, and the ability of NO to modulate such a wide range of signalling pathways allows for the possibility that dysregulated s-nitrosation could contribute to the pathophysiology of an equally wide range of diseases.

We have recently determined that the impact of BH4 on cellular redox signalling in endothelial cells and animal models, where abolition of BH4 leads to increased cellular generation of superoxide^16–19^. Moreover, we also demonstrated that a proportion of this superoxide is produced directly from uncoupled eNOS, and that BH4 plays an important role in the mitochondria^20^. While these studies show that the molecular and cellular mechanisms through which BH4 exerts its effects on eNOS are now well established, the downstream impact of these regulatory mechanisms on eNOS-dependent signalling, and the identification of the resulting target proteins remain unknown. The aim of this study was to explore the impact of BH4 deficiency on cellular redox balance, and to discover novel proteins and pathways that are altered through BH4-dependent NO signalling by S-nitrosation. We uncovered NO modifications on critical components of the ubiquitin-proteasome system with possible widespread implications in NO-mediated cellular processes.

## Methods

### Cell Culture

sEnd.1 murine endothelial cells and Tet-regulatable GCH cells were cultured in DMEM (Invitrogen), supplemented with 10% FBS, glutamine (2 mmol/litre), penicillin (100 units/ml), and streptomycin (0.1 mg/ml). We used NIH 3T3 murine fibroblasts stably transfected with a *tet*-off transactivator construct. In the presence of doxycycline, binding of the transactivator is blocked, and gene expression is prevented. These initial 3T3-*tet*-off cells, previously shown to express neither eNOS nor GTPCH and also confirmed to be devoid of neuronal NOS, inducible NOS, and eNOS protein, were stably transfected with a plasmid encoding hemagglutinin (HA) antigen-tagged-human GCH1 under the control of a tetracycline-responsive element, Individual colonies were isolated and analyzed for GCH1 expression and a cell line, termed “GCH cells,” was established from expansion of a single colony. GCH cells were stably transfected with a plasmid encoding a human eNOS-eGFP fusion protein and clones were picked. All of the cell lines underwent three rounds of clonal selection. GCH cells were maintained in medium containing Hygromycin B (200 μg/ml) and Geneticin® (200 μg/ml). NIH 3T3 murine fibroblasts were stably transfected with a Tet-Off transactivator construct, as previously described. Doxycycline (1 μg/ml) was added to cell culture media to abolish transcription of *Gch1* mRNA where appropriate as previously described^7^.

### Gch1 Knockdown by RNA Interference

*Gch1*-specific, ON-TARGETplus SMARTpool siRNA were purchased from Dharmacon Thermo Scientific. The siRNA were used as a pool of four specific siRNA duplexes with the following sequences^6,7^: Duplex 1, GGUAGAAUGCUAAGUACGU; Duplex 2, CGAGAAGUGUGGCUACGUA; Duplex 3, GAGAAGGGAGAAUCGCUUU; and Duplex 4, AGUAGUGAUUGAAGCGACA. 24 h prior to transfection, sEnd.1 cells were seeded into 6-well plates. Cells were then transfected with *Gch1*-specific siRNA (100 nmol/litre), or nonspecific (NS) pooled duplex negative control siRNA (100 nmol/litre), using DharmaFect1 transfection reagent (Dharmacon). Cells were cultured for 72 h and gene silencing was detected by analysis of GTPCH protein expression by means of Western blotting using GTPCH-specific antibodies.

### Biopterin Quantification by HPLC with Electrochemical Detection

BH_4_, BH_2_, and biopterin levels in cell and mitochondrial lysates were determined by HPLC followed by electrochemical and fluorescent detection, as described previously. Briefly, the cells were grown to confluency and harvested by trypsinisation. Mitochondria from confluent T175 flasks were isolated using the Qproteome Mitochondrial Isolation Kit (Qiagen). Sample pellets were resuspended in PBS (50 mmol/litre), pH 7.4, containing dithioerythritol (1 mmol/litre) and EDTA (100 μmol/litre) and subjected to three freeze-thaw cycles. Following centrifugation (15 min at 17,000 × g, 4 °C), the samples were transferred to new, cooled microtubes and precipitated with ice-cold extraction buffer containing phosphoric acid (1 mol/litre), trichloroacetic acid (2 mol/litre), and dithioerythritol (1 mmol/litre). The samples were vigorously mixed and then centrifuged for 15 min at 17,000 × g, 4 °C. The samples were injected onto an isocratic HPLC system and quantified using sequential electrochemical (Coulochem III, ESA Inc.) and fluorescence (Jasco) detection. HPLC separation was performed using a 250 mm, ACE C-18 column (Hichrom) and mobile phase comprising of sodium acetate (50 mmol/litre), citric acid (5 mmol/litre), EDTA (48 μmol/litre), and dithioerythritol (160 μmol/litre) (pH 5.2) (all ultrapure electrochemical HPLC grade), at a flow rate of 1.3 ml/min. Background currents of +500 μA and −50 μA were used for the detection of BH_4_ on electrochemical cells E1 and E2, respectively. 7,8-BH_2_ and biopterin were measured using a Jasco FP2020 fluorescence detector. Quantification of BH_4_, BH_2_, and biopterin was made by comparison with authentic external standards and normalised to sample protein content^7,21^.

### Western Blotting

Cell lysates were prepared by homogenisation in ice-cold CelLytic™ M buffer (Sigma) containing protease inhibitor cocktail (Roche Applied Science). Lysates were centrifuged at 17,000 × g for 10 minutes at 4 °C, and samples were prepared using LDS sample buffer (Invitrogen). Western blotting was carried out using standard techniques with anti–GAPDH, –GTPCH, –eNOS, –Rluc8.1, –Rluc8.2, –Ub, –E2N, –E2V1 antibodies.

### The enrichment and detection of protein-SNO by the biotin switch technique

Cysteine-bound NO was replaced with biotin using an adaptation of the Biotin Switch technique as described previously^22,23^. Following trypsinolysis, pulled down peptides were then detected using mass spectrometry, and the target proteins by Western blotting using specific antibodies as described below. We detected 543 altered peptides in 477 proteins, with the requirement that every SNO peptide contained at least one cysteine.

### Organomercury capture of SNO proteins

Phenylmercury resin (kindly provided by Professor Harry Ischiropoulos and Dr Paschalis Doulias, University of Pennsylvania, USA) was used to capture NO-Cys containing proteins, using mass spectrometry as previously described. Proteins bound to the resin were then eluted using Beta-mercaptoethanol and specific target proteins of interest probed by Western blotted using specific antibodies^24,25^.

### SNO, Nitrite and Nitrate quantification by NOA

Following preincubation for 1 h in the presence or absence of L-monomethylarginine (100 μmol/liter) in Krebs-Henseleit buffer (consisting of 120 mmol/liter NaCl, 4.7 mmol/liter KCl, 1.2 mmol/liter MgSO_4_, 1.2 mmol/liter KH_2_PO_4_, 2.5 mmol/liter CaCl_2_, 25 mmol/liter NaHCO_3_, and 5.5 mmol/liter glucose), the cells were exposed to A23187 (1 μmol/liter) for 30 min. Total nitrite and nitrate accumulation was measured using the CLD88 NO analyzer (Ecophysics), following published protocols for SNO and NOx.

### Creation of S-nitrosation Biosensors

Rluc8.1 and 8.2 fragments were PCR-amplified using the rluc8 plasmid as a template (a gift from Prof. Sanjiv Gambhir, Stanford University)^26^. The rluc8 PCR fragments were cloned into pcDNA3. Gateway destination plasmids were then created by cloning the vector conversion kit (Invitrogen) into the rluc8.1 and rluc8.2 vectors, as previously^17^. For the creation of stable cell lines, this destination cassette was cloned into the pIRES-puro and Neo plasmids (Clontech). pENTR clones encoding our genes of interest were either created by PCR or purchased from Geneservice or Thermo Fisher. To create expression plasmids, pENTR clones were recombined with the destination vectors using LR clonase according to the instructions of the manufacturer. Recombinant Rluc8 expression plasmids and a LacZ-encoding control plasmid were then transfected into sEnd.1 or eNOS/GCH cells and grown on white 96-well plates using FuGENE HD (Roche). Final constructs were pExp-Xpo1-Rluc8.1N-pires-puro, and pExp-Rev-Rluc8.2C-pires-neo. We placed our rluc8.1 and rluc8.2 halves onto either end of Xpo1 and REV, respectively.

### Detection of Biosensor Luminescence by Protein Fragment Complementation Assay

24 h following transfection, cells were treated with drugs as indicated. Cells were then washed with PBS, and reconstituted Rluc8 activity was measured using benzyl-coelenterazine (Nanolight) on a BMG Polarstar plate reader (5-s read time). Cells were lysed by adding LacZ lysis buffer (120 μM TrisPO4 (pH 7.8), 10 mM 1,2-cyclohexylenedinitrilotetraacetic acid, 30% glycerol, and 1% Triton X-100) to each well, and LacZ activity was measured using ortho-nitrophenyl-β-galactopyranoside. Rluc8 readings were normalized using LacZ activity.

### Proteomics analysis

Cell pellets were dissolved in 6 M urea in 50 mM ammonium bicarbonate. Dithiothreitol (10 mM) was added and samples were incubated at 60 °C for 45 minutes. Samples were diluted 1:10 in 50 mM ammonium bicarbonate and sequencing grade modified trypsin (Promega) was added (1:50 w/w) for overnight digestion. PMSF (0.5 mM) stopped the reaction and samples were dried down in a vacuum concentrator (Speedvac, Thermo Scientific) and resuspended in acetonitrile (2%) and TFA (0.1%). Samples were desalted using ZipTipC18 pipette tips (Millipore), vacuum concentrated and reconstituted in acetonitrile (2%) and TFA (0.1%), before mass spectrometric analysis.

### Mass spectrometry analysis

Liquid chromatography tandem mass spectrometry (LC-MS/MS) was performed using an Orbitrap Velos mass spectrometer, coupled with a Waters nanoAquity UPLC essentially as described previously^27^. In brief, injected samples underwent online desalting using a Trap column Symmetry C18, 180 µm × 20 mm, 5 µm particle, Waters). For separation, a BEH C18 column (75 µm × 250 mm, 1.7 µm particle, Waters) was used with a flow rate of 250 nl/min over 60 minutes and a gradient of 3–40% acetonitrile 0.1% Formic acid. Survey scans were acquired in the orbitrap with a resolution of 60,000 at 400 m/z between 300 and 2000 m/z for up to 100 ms with an ion target of 1E6. Selected precursors were picked above a threshold of 5E2 counts for MS/MS and excluded for 30 s after sampling. MS/MS spectra were acquired in the ion trap with a maximum accumulation time of 100 ms and an ion target of 5E4 counts. CID fragmentation was performed with a normalized collision energy of 35. Experiments were conducted in biological triplicates and all sample analysed in a single sample batch.

### Proteomic data analysis

For label free quantitation raw data were imported into Progenesis QI (Waters) with default settings. MS2 spectra were identified with Mascot v 2.4 (Matrix Science) by searching Uniprot/Swissprot (retrieved 07/09/2012) with taxonomy Mus musculus, using 10ppm and 0.6 Da peptide mass/fragment mass tolerances. Allowance was made for 1 missed cleavage site. Biotin-HPDP (C), DeStreak (C), Deamidation (N/Q) and Oxidation (M) were set as variable modifications. Data was exported for integration in Progenesis with a peptides false discovery rate of 1%. Furthermore, spectra with a peptide score <20 were discarded. Peptide abundances were mean normalised to identified features only. Biotinylated proteins in the *Gch1* knockdown group were subtracted from those that appeared in the NS group, leaving our group of interest where the appearance of detectable nitrosation was dependent on *Gch1* expression and the presence of BH4.

### Gene ontology overrepresentation analysis of BH4 enriched proteins

BH4 enriched proteins were submitted to PANTHER (version 12) for overrepresentation analysis against the complete Gene Ontology biological process, molecular function and cellular component classes. Significantly overrepresented (Bonferroni corrected p < 0.05) terms were displayed in the Enrichment Map plugin (version 2.2.1) for Cytoscape (version 3.5.1) using a Jaccard coefficient of 0.45. Nodes were automatically clustered with the AutoAnnotate plugin using MCL clustering with the similarity coefficient used for edge weighting. All single, unconnected nodes were removed to create the final enrichment map.

### Molecular Modeling of the nitrosation sites of Ubiquitin E2N and 26S proteasome subunits

Figures were produced using PyMOL, using the PDB codes 5AIT, 5L4G and 5L4K.

### E2 conjugating enzyme activity assay

E2 activity was assessed using recombinant protein as previously described^28^. Briefly, E1 (0.3 µM), E2N (2.5 µM), E2V1 (2.5 µM) and CHIP (1.0 µM) were mixed in the reaction buffer (50 mM Hepes pH 7.6, 100 mM NaCl_2_ and 5 mM MgCl_2_) and incubated in the presence of ATP (2 mM) and ubiquitin (100 µM) for 1 h at 37 °C. Free ubiquitin chain formation catalysed by CHIP was quantified by Western blotting using anti-ubiquitin and anti-CHIP specific antibodies.

### Statistical Analysis

Data are expressed as mean ± SEM. Comparisons between NS and GCH siRNA were made by the unpaired Student’s *t*-test. Experiments testing multiple treatments between cell type and knockdown were compared by two-way ANOVA, with post-hoc tests applied to test for significance between knockdown and treatments, as outlined in the figure legends. *P* < 0.05 was considered statistically significant.

## Results

### Gch1 knockdown-induced BH_4_ deficiency determines NO metabolite accumulation

**Figure 1:**
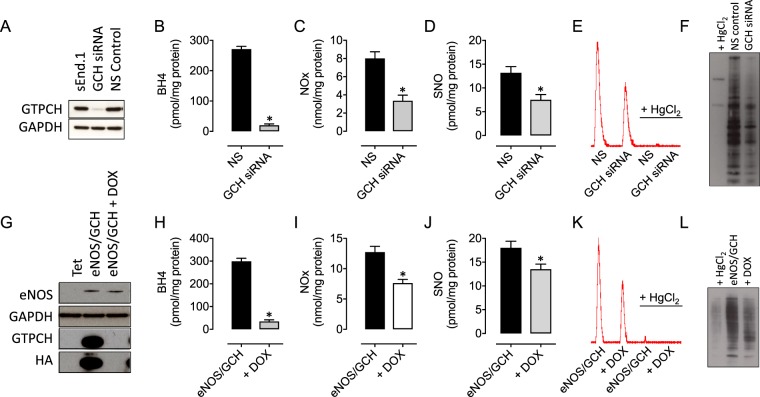
Depletion of endothelial BH4 results in attenuated accumulation of protein s-nitrosothiols. Exposure of sEnd.1 endothelial cells to GCH1-specific siRNA (**A**) decreases GTPCH protein expression, (**B**) BH4 levels, and (**C**) markedly attenuates the accumulation of NO metabolites including NOx and s-nitrosothiols (**D**), and NO analyser traces in (**E**), compared to NS scrambled control siRNA treated cells (*P < 0.05, n = 4/6). Protein s-nitrosation is also strikingly diminished in GCH-siRNA vs NS scrambled control cells, as demonstrated using the biotin switch technique, compared to HgCl_2_ treated negative control cells (**F**), n = 3. Comparable findings were shown in an alternative model of BH4 deficiency and eNOS uncoupling, using tet-regulatable eNOS/GCH cells in the presence or absence of doxycycline (**G**–**L**), *P < 0.05, n = 6–8).

We first tested the effect of targeting *Gch1* gene expression, through RNA interference, on the knockdown of GTPCH protein and examined the subsequent effects on cellular BH_4_ levels. Transfection of sEnd.1 cells with *Gch1*-specific siRNA lead to a substantial decrease in GTPCH protein, as detected by Western blotting (Fig. 1A). The depletion of GTPCH was paralleled by a >90% decrease in cellular BH_4_ levels (Fig. 1B). This depletion of BH_4_ was sufficient to cause a striking 50% decrease in the accumulation of NOx, and attenuate SNO accumulation significantly (Fig. 1C–E). A decreased fraction of S-nitrosated proteins was also detected using the ‘Biotin Switch’ technique (Fig. 1F). These data were replicated in a complementary system where GTPCH and BH4 production is attenuated using doxycycline in tetracycline-regulatable cells expressing eNOS (Fig. 1G–L). Importantly, again abolition of BH4 lead to a marked decrease in s-nitrosation, demonstrated using biochemical and biotin switch-based techniques.

**Figure 2:**
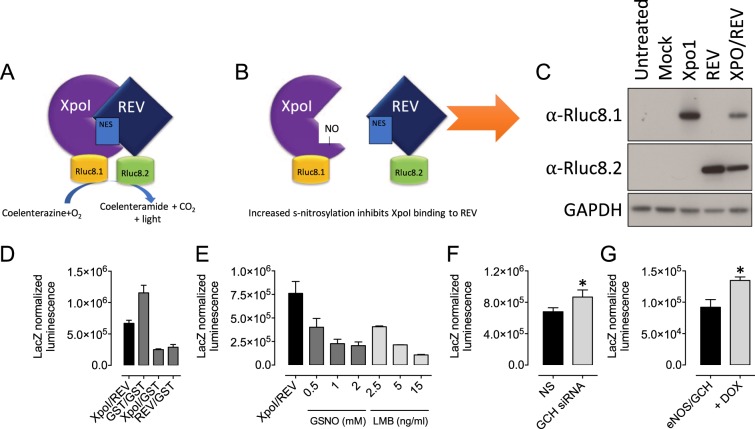
These changes in SNO signalling are quantifiable using a novel split-renilla based luciferase biosensor. A split renilla biosensor was created based on the SNO-dependent interaction of Xpo1 and REV as described previously^17^ (**A**) Each half of renilla (8.1 and 8.2) was cloned onto the C-terminus of each protein. (**B**) When a critical cysteine in Xpo1 is nitrosated, the Xpo1-REV interaction is abolished. (NES = nuclear export signal). (**C**) Overexpression of the individual proteins, lagged with Rluc8.1 were detected using Western blotting using specific antibodies, and (**D**) Co-overexpression of Xpo1–8.1 and REV-8.2 results in a luminescence signal, as detected using a luminescent plate reader, which is significantly greater than that of Xpo1/GST and REV/GST negative control overexpression. GST/GST = positive control. (**E**) This SNO-dependent interaction of XPO1 and REV responds to a dose response of GSNO and LMB. A decreased signal is observed in the presence of either GSNO (0.5–2 mM) or LMB (2.5–15 ng/ml). When these constructs were expressed in either (**F**) sEnd.1 endothelial cells in the presence or absence of GCH1-specific siRNA, or (**G**) eNOS/GCH tet-regulatable cells plus doxycycline, an increased interaction was detected indicative of decreased cellular SNO levels. (n = 5/6, *P < 0.05).

Having shown for the first time that BH4 determines cellular s-nitrosothiol levels and protein s-nitrosation, it was important to use an alternative method to overcome the limitations of the ‘biotin switch’ technique. To this end, we developed a novel biosensor on the basis of the reconstitution of *Renilla* luciferase as depicted in Fig. 2, as we have previously successfully developed to detect eNOS dimerization^17^. The SNO biosensor was created from the rluc8 construct as described under “Experimental Procedures.” We based the biosensor on the interaction of the karyopherin chromosomal region maintenance 1 (CRM1/XPO1) protein with REV, a nucleocytoplasmic shuttling HIV protein. This interaction has previously been shown to be dependent on the s-nitrosation of XPO1, where XPO1-dependent nuclear export is repressed by NO (Fig. 2A,B). We first characterized the biosensor by Western blotting and luminometry. Following transient transfection with either the experimental Xpo1- or REV-Rluc fusion constructs or a GST control biosensor, HEK293 cells were shown to express either Xpo1-Rluc8.1, REV-Rluc8.2, GST-Rluc8.1, or GST-Rluc8.2, where appropriate, using Rluc8.1- or Rluc8.2-specific antibodies (Fig. 2C). We placed our rluc8.1 and rluc8.2 halves onto the C-terminus of either Xpo1 or REV. Cells were then transfected with both constructs and luminescence was measured by protein fragment complementation assay. During the setup and initial characterization of our system, we tested the ability of luciferase to generate a detectable signal when Xpo1-rluc8.1 and REV-rluc8.2 were co-overexpressed, compared to the negative control pairings of Xpo1-Rluc or REV-Rluc with GST-Rluc. Luciferase luminescence was only detectable in cells expressing both Xpo1 and REV, or both tagged GST constructs (Fig. 2B). The specificity of the interaction of Xpo1 and REV was then tested using the NO donor, GSNO, and the specific inhibitor of XPO1, Leptomycin B (LMB). Cells were treated with GSNO or LMB for 4 h, and the XPO/REV interaction assessed by luminescence. In cells expressing both the Rluc8.1 and Rluc8.2 fusion proteins, a luminescence signal was detectable. Upon exposure of the cells to a titration of doses of GSNO, the detectible luminesce signal decreased accordingly; the interaction of XPO1 and REV, was decreased by 70% upon exposure of the biosensor to GSNO (1 mM). The same result was achieved upon exposure of the biosensor-treated cells with LMB (Fig. 2E). This suggests that GSNO is disrupting the interaction of XPO1 and REV in a NO dependent manner, disassociating the complex to a greater extent as the concentration of GSNO increases; the control cells, treated with LMB show the same trend, where binding of LMB to the active site cysteine in XPO1 decreased luminescence by 2-fold at 2.5 ng/ml, and approaching 10-fold at 15 ng/ml for 30 min. In order to address the impact of BH4 on protein S-nitrosation, we then overexpressed the luciferase biosensor in murine endothelial cells concomitant with siRNA that target *Gch1*. We also expressed the biosensor in our tetracycline regulatable eNOS/GCH cell line, and demonstrate that we were able to detect a significant increase in luciferase signal, indicative of diminished protein S-nitrosation in both models of BH4 deficiency (Fig. 2F,G, *P < 0.05, n = 4). These data using this unique biosensor approach further support our finding that BH4 level modulates protein-SNO in endothelial cells.

### BH4 has wide ranging impact on a range of signalling pathways via protein s-nitrosation

**Figure 3:**
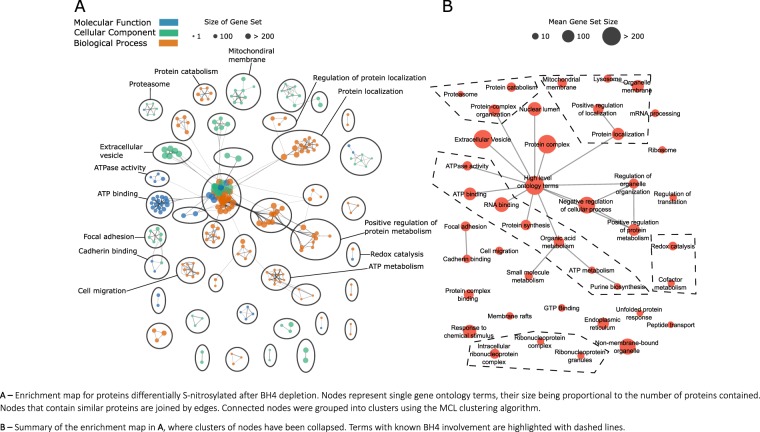
Functional analysis of the BH4-dependant s-nitrosoproteome reveals that the impact of BH4 on cellular signalling by NO is widespread. (**A**) Enrichment map for proteins differentially S-nitrosylated after BH4 depletion. Nodes represent single gene ontology terms, their size being proportional to the number of proteins contained. Nodes that contain similar proteins are joined by edges. Connected nodes were grouped into clusters using the MCL clustering algorithm. (**B**) Summary of the enrichment map in A, where clusters of nodes have been collapsed. Terms with known BH4 involvement are highlighted with dashed lines.

We used PANTHER’s overrepresentation analysis to determine the global effects of BH4 deficiency induced by knockdown of GTPCH1. S-nitrosylated proteins were captured from cell lysates using the biotin switch method. Functional analysis of the SNO proteome (Fig. 3) after BH4 depletion reveals BH4 dependant S-nitrosation in a wide range of pathways and cellular compartments. These pathways include both known BH4 functions (e.g. redox signalling, mitochondria) and potentially novel pathways such as the ubiquitin-proteasome pathway and cell-cell/cell-matrix adhesion. However, it is important to note that although these data demonstrate enriched localization of Cys-NO abundance in particular compartments, they do not necessarily imply regulation of those proteins by NO modification, without further validation.

### Modulation of ubiquitin-proteasome pathway by BH4-dependent s-nitrosation

**Figure 4:**
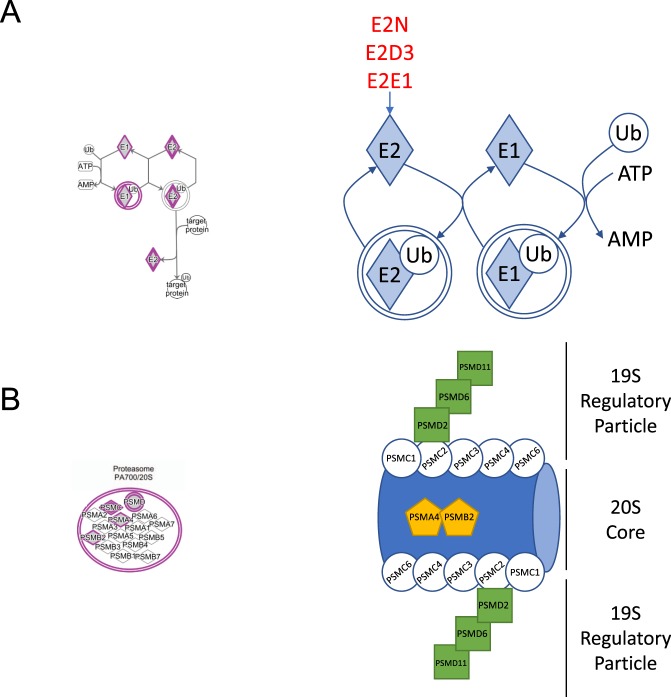
BH4 modulates the nitrosation of key proteins in the Ubiquitin-Proteasome system. (**A**) Qiagen ingenuity pathways analysis (IPA) uncovers ‘protein ubiquitination’ and (**B**) the ‘26S Proteasome’ as the top pathway demonstrated to be altered in a BH4- and NO-dependent manner, with specific targets highlighted in Blue. (**C**) Specific NO targets (E2N, E2D3 and E2E1) within the E2 conjugating enzymes are illustrated in schematic form. (**D**) IPA analysis also suggests that BH4-dependent modification by NO is important to the function of the proteasome multi-catalytic complex (the 20S core and 19S regulatory particle), and all modified subunits are depicted in the schematic.

**Figure 5:**
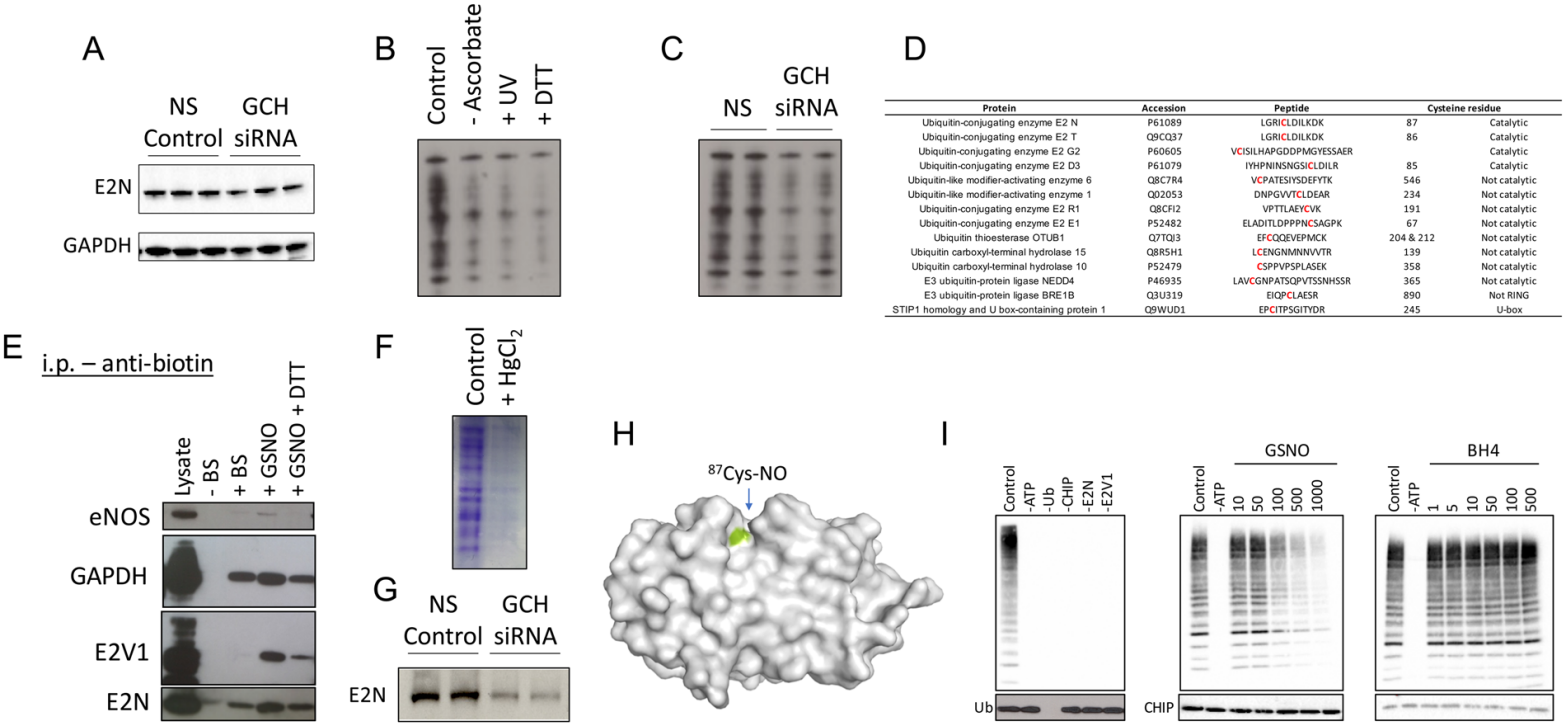
E2N is a target for BH4-dependent signalling via NO. BH4 deficiency alters NO/Redox balance and subsequent pottranslational modification of targets by NO. (**A**) Important controls are shown, validating the specificyt of the biotin switch for Cys-NO, where detectable signal is significantly attenuated in the absence of ascorbate, and following pre-reduction by exposure to UV and DTT. (**B**) Decreased abundance of nitrosated proteins are detected in GCH- vs Non-specific siRNA treated endothelial cells. (**C**) Cysteine residues within E2 conjugating enzymes are profoundly modified, and a significant number of those cysteine-containing peptides are catalytic cysteines that would be predicted to modify protein activity, and these changes occur independently of changes in total E2N protein shown by Western blotting using a E2N–specific antibody (**D**). (**E**) Protein lysates underwent the biotin switch procedure following exposure to either GSNO or GSNO and DTT. Detection of biotin-Cys using specific antibodies following pull down, clearly demonstrates that E2N is susceptible to nitrosation. (**F**) A complimentary method where SNO proteins are captured using organomercury supports the biotin switch findings was used to support the biotin switch findings; pre-exposure to HgCl_2_ abolished detectable SNO protein, and (**G**) E2N is clearly detectable in NS, but is markedly decreased in GCH-specific siRNA treated endothelial cells. (**H**) Using molecular modelling of the Cys87-NO in E2N by PYMol, it can be seen that Cys87 is exposed in a binding pocket (shown in Green). (**I**) The effect of nitrosation on the activity of E2N was studied using a recombinant protein activity assay. Recombinant proteins were incubated in the presence or absence of GSNO and BH4, as described in the Experimental Procedures. Polyubiquitination was only detected in the presence of all components of the assay, and did not occur when any one protein or substrate was omitted. The activity of E2N was inhibited by GSNO in a dose-dependent manner, and remained unchanged when exposed to BH4, thus demonstrating that the effects of BH4 are NO dependent, and not through unexpected direct effects of BH4 on protein activity (n = 3).

The proteomics screen for protein s-nitrosation in endothelial cells devoid of BH4 reveals that the enrichment of certain cellular compartments such as the mitochondria for the process of s-nitrosation. Moreover, IPA pathway analysis identified protein ubiquitination as an enriched pathway that was suggested to be modulated in a BH4- and NO-dependent manner, shown in Table 1 and Fig. 4. These findings are striking as they implicate BH4 in a number of cellular processes including the regulated and targeted proteasomal degradation of substrate proteins. In order to validate, and further advance this pathway analysis result, we focused on the individual members of the ubiquitination pathway, using the biotin switch technique. Important controls confirm the specifity of the assay for Cys-NO (Fig. 5A,B), and mass spectrometry analysis revealed that several members of the pathway were s-nitrosated at various cysteines (Fig. 5C). Four of these peptides (all E2 enzymes), were discovered to be nitrosated within the proteins active site, specifically on the catalytic cysteine. The potential impact of these modifications is summarized in the schematics for the E2 conjugating enzymes, and proteasome, outlined in Fig. 4.

**Table 1:** Qiagen Ingenuity Pathway Analysis identifies several pathways to be enriched, when comparing those hits detected in BH4 replete, verses deplete endothelial cells.

Canonical Pathways	−log (p-value)	Ratio
Protein Ubiquitination Pathway	11.1	0.11
EIF2 Signaling	11	0.118
Signaling by Rho Family GTPases	9.91	0.105
RhoGDI Signaling	7.7	0.11
Cardiac Hypertrophy Signaling	3.93	0.0681
Chemokine Signaling	3.66	0.113
Endothelin-1 Signaling	3.39	0.0695
Mitochondrial Dysfunction	3.21	0.0702
Hypoxia Signaling in the Cardiovascular System	3.14	0.108
Tight Junction Signaling	2.76	0.0659
TCA Cycle II (Eukaryotic)	2.76	0.174
Wnt/β-catenin Signaling	2.72	0.0651
Glutathione Redox Reactions II	2.51	0.5
Proline Biosynthesis I	2.51	0.5
NF-κB Signaling	2.51	0.0611
Tetrahydrofolate Salvage from 5,10-methenyltetrahydrofolate	2.3	0.4
NRF2-mediated Oxidative Stress Response	2.28	0.057
VEGF Signaling	2.01	0.068
eNOS Signaling	2.01	0.0581
Oxidative Phosphorylation	1.88	0.0642

We hypothesized that modification by NO at these sites would potentially have striking effects on their function. Within this pathway, we were particularly interested in Ubiquitin conjugating enzyme E2N, where the BH4-dependent nitrosation occurred at cysteine 87 (Fig. 5C). Pulldown of SNO proteins using the Biotin Switch technique, followed by Western blotting using an anti-biotin antibody revealed that s-nitrosation of E2N was present in endothelial cell lysates, and that this nitrosation was elevated after exposure of the lysate to GSNO, along with eNOS, GAPDH and E2V1. Pre-reduction with DTT attenuated the abundance of SNO-E2N. (Fig. 5E). Confirmation of E2N as a target for modification by NO, and its subsequent modulation by BH4 was also demonstrated using the complementary technique of organomercury resin assisted capture and Western blotting. Importantly, HgCl_2_ pretreatment of cell lysates demonstrated the specificity of the assay for Cys-NO and S-nitrosation of E2N was clearly detectable in control cells. This specific modification of E2N protein by NO was attenuated in cells that produce less NO, due to the knockdown of GTPCH by siRNA (Fig. 5G). This modification occurred independently of a change in total protein level, and was shown to be in the active site pocket highlighted in Fig. 5H.

Having identified new BH4-dependent pathways and a potential role for BH4 in protein ubiquitination, our next aim was to establish whether s-nitrosation of Cys87 did indeed alter the function of E2N. Addressing the activity of individual ubiquitin enzymes in cell culture systems is not possible, so to alleviate this limitation, we used a recombinant protein activity assay. This E2 activity assay was based on the formation of free ubiquitin chains, catalysed by CHIP, where E1, CHIP, E2V1 and E2N proteins were combined, incubated for 1 h and ubiquitination was detected by Western blotting using an anti-ubiquitin antibody, as outlined in the *Experimental Procedures*. The activity assay was carried out in the presence of either GSNO or BH4 to mimic the effect of s-nitrosation, and to compare the effects of NO donation by GSNO and rule out any unexpected effect of a direct action of BH4 itself. Ubiquitination was detected, when all proteins where incubated in the presence of ATP. Ubiquitination was not detected when any single protein, or ATP was not included (Fig. 5F). Ubiquitination was dose-dependently decreased when GSNO was added, with ubiquitination almost abolished following exposure to GSNO (1 mM). In contrast, the enzyme activity assay remained unaltered when BH4, rather than GSNO was included, suggesting that the effects of BH4 on protein function are mediated through NO, not by direct modulation of E2 activity by BH4. Importantly, the impact of GSNO on polyubiquitination occurred independent of any changes in E1-E2N thio-ester formation (Supplemental Fig. 1), which would support the lack of E1 modification by NO in our mass spectrometery dataset.

**Figure 6:**
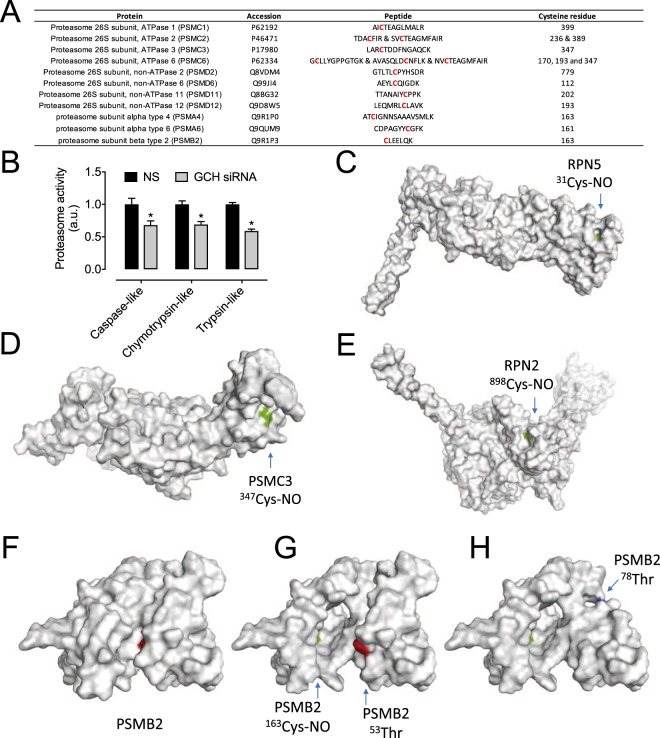
BH4 positively regulates the activity of the proteasome. Posttranslational modification of proteasome targets by NO. (**A**) Cysteine residues within subunits of the 26S proteasome are profoundly modified in a BH4 dependent manner. (**B*–*D**) PSMC6, RPN2 and RPN5 subunits are all demonstrated to be altered at specific sites, where the Cys side chains are shown in green. Human models have been presented. (**E**) sEnd.1 endothelial cells were exposed to GCH-specific, or NS control siRNA, and proteasome activity was assessed using a Promega proteasome activity assay as in the Experimental Procedures. Knockdown of BH4 levels, and subsequent NO/redox imbalance using siRNA, significantly inhibited proteasome activity (n = 4, *P < 0.05). (**F**) Nitrosation sites in PSMB2 (shown in Green) are only visible upon processing of the protein where cleavage at ^53^Thr and ^78^Thr occurs during proteasome maturation as shown in red and blue, respectively (**G**,**H**).

It was also found that the proteasome pathway was significantly altered in a BH4 dependent manner. Indeed, the proteasome subunits are a significant part of the pathway shown in Fig. 4. Mass spectrometry analysis revealed S-nitrosation of cysteine residues within proteasomal subunits (PSMC, PSMD, and catalytic subunits PSMA4 and PSMB2) (Fig. 6A). This is consistent with what has been observed in vascular smooth muscle cells that were directly treated with NO^29^. To test the functional impact of this finding, we used a fluorescent assay to measure the three classical activities of the complex. Indeed, exposure of sEnd.1 endothelial cells to GCH-specific siRNA resulted in significant attenuation of chymotrypsin-, trypsin-, and caspase-like activities (Fig. 6B). We then used a PyMol molecular modelling approach to gain some insight into the mechanism by which modification by NO would lead to the inhibition of proteasome activity (Fig. 6C–E). In the case of PSMB2, we examined the location of these BH4-dependent NO-Cys residues relative to the catalytic N-terminal Thr residue (Fig. 6F–H), and show that in the catalytic beta subunit PSMB2, NO-Cys163 is to distal from N-Thr53/78 to allow direct interaction with NO, therefore suggesting that inhibition of catalysis appears to happen in an indirect fashion. In addition, a number of NO-Cys-modifications have also been detected in the regulatory ATP (PSMD1, PSMC2, PSMC6) and non-ATP subunits (PSMD2, 6, 11, 12), providing the basis for further potential modulation of function (Fig. 6).

Taken together, these data demonstrate for the first time that the impact of BH4 is far wider than originally anticipated, and that BH4 via s-nitrosation of target proteins, can dictate the function of downstream cellular pathways, not previously linked with BH4 or NO.

## Discussion

In this study, we reveal the impact of altered BH4 production on the redox effector functions of the eNOS/BH4 redox ‘hub’. We demonstrate that altering BH4 level alone is sufficient to perturb the production of NO and subsequent protein SNO formation, and use a combined proteomic and novel biosensor approach, (with validation by organomercury enrichment) to show for the first time that this change in BH4 can have a drastic influence on the identification and magnitude of downstream posttranslational signalling by S-nitrosation. We then probed one of the most significantly altered pathways identified from the proteomics gene ontology data analysis, that of protein ubiquitination, and used a biochemical approach to validate the effect of BH4-dependent s-nitrosation on its activity, implied by the unbiased proteomics screen, and corroborated by two different methods. Furthermore, we show that SNO modification of a critical cysteine residue in a candidate E2 conjugating enzyme does indeed interfere with its function, along with reducing the activity of the proteasome. This study clearly demonstrates the importance of BH4 as a modulator of redox signalling and indicates that BH4 and eNOS, acting as a signalling ‘hub’ may modulate a plethora of new biochemical pathways.

The idea that modification of protein function by nitrosation is involved in cardiovascular homeostasis and disease is not novel, however the notion that BH4 availability determines ubiquitous downstream signalling via this posttranslational mechanism has not been addressed until now. Having been proven to occur in a plethora of proteins with roles in cardiovascular homeostasis, nitrosation now explains the wide range of cellular effects of NO. For example, nitrosation of HIF-1 alpha increases VEGF production and myocardial capillary density in the heart, while addition of NO to a critical cysteine in Caspase 3 opposes apoptosis and preserves endothelial function. In separate experiments, BH4 has also been widely implicated to have similar roles, being required for the effects of VEGF, and having antiapoptotic functions in the endothelium^30–32^. In addition, aberrant S-nitrosation is common in cardiovascular disease and can at least in part explain the dysfunctional actions of the ryanodine receptor in cardiac arrythmogenesis^33,34^ as well as in pulmonary hypertension^35^. In both of these conditions, as well as many others, supplementation of BH4 has been shown to be beneficial in animal models, predominantly through restoring NO production and normal NO signalling.

The subcellular compartment absolutely determines the targets of redox signalling, along with the type and source of the signalling moiety. Interestingly, our data expose the mitochondria as a key subcellular compartment where BH4-dependent nitrosation takes place, highlighted in Table 1, and also presented in Fig. 3. This is consistent with the large proportion of mitochondrial proteins known to be modulated by NO; such as complex I, where deactivation by NO slows the reactivation of mitochondria during the crucial first minutes of the reperfusion of ischemic tissue, thereby decreasing ROS production, oxidative damage and tissue necrosis. Our recent report studying the ‘non-canonical’ roles for BH4 was the first to suggest that BH4 dictates redox balance in the mitochondria. Specifically, BH4 depletion resulted in striking production of ROS from the mitochondria, changes in antioxidant protein expression, and marked alterations of the cellular proteome and metabolome signature of the endothelial cell in the absence of BH4. These changes in cellular biochemistry, metabolism and the changes in posttranslational signalling presented here, underlie the importance of BH4 as a regulator of cellular function.

The aim of this study was to uncover new mechanisms in which BH4 may play a role, helping us understand the wider effects of BH4, and elucidating new mechanisms we can exploit in the design of future drug treatments. To this end, the most prominent finding from the unbiased proteomics screen in these data was the requirement of the ubiquitin-proteasome system for BH4. Indeed, Ingenuity Pathway Analysis exposed this pathway as the most altered by BH4-dependent nitrosation, as detected using both the biotin switch and organomercury capture techniques. Importantly, BH4 is also a critical cofactor for the aromatic amino acid hydroxylase enzymes such as phenylalanine and tyrosine hydroxylase, and therefore BH4 plays important roles in the synthesis of neurotransmitters such as dopamine and noradrenaline. The data presented in this study would suggest that effects of BH4 insufficiency on protein s-nitrosation of key enzymes within the ubiquitin proteasome pathway occur in an NO-dependent manner, independent of these other cofactor roles.

Ubiquitination is an enzymatic posttranslational modification process that occurs on proteins based on the ligation of ubiquitin (an 8.5 kDa protein) on a target protein lysine residues. This covalent attachment of ubiquitin on lysine residues requires the coordinated reaction of three enzymes: E1 enzyme, which promote ubiquitin adenylation required for its covalent binding to the cysteine residue located at the E1 active site; E2 (ubiquitin conjugating enzymes) that accept ubiquitin from the ubiquitin ‘loaded’ E1; and E3 ubiquitin ligases that are required for target recognition and final transfer the ubiquitin to the protein for targeted for degradation. Protein ubiquitination is therefore central to the mechanism underlying protein turnover and quality control, as it is responsible for the redirection of damaged/unfolded proteins towards the proteasome to be degraded. It is now commonly accepted that ubiquitination also plays critical roles in the turnover of aged organelles, and cellular processes and signalling which include antigen processing, apoptosis, cell cycle, DNA transcription, and repair. The specificity of the Ubiquitin-proteasome system is dictated by the E3 protein, which selects the substrate, and there are hundreds of E3 ligases. Our data presented herein suggests that BH4 is central to the activity of E2 conjugating enzymes as exemplified by our discovery that Cys87 on Ubc13 is modified and most likely interferes with the formation of ubiquitin thioester intermediates before the transfer to the cognate E3 ubiquitin ligase (Fig. 5C). This implies that BH4 may subsequently be important in the wide range of pathways that contain the proteins targeted for K63-polyubiquitylation that may be regulated by NO. If we hypothesize that one E2 conjugating enzyme controls the passing of ubiquitin from multiple E3 ligases, each to multiple target proteins, then the potential impact for regulation of the ubiquitin-proteasome system by BH4 is vast.

Beside the effects on ubiquitin conjugating system, NO has been shown to directly interfere with the activity of the proteasome complex. Our data demonstrating that BH4-dependent nitrosation affects proteasome catalytic activity is supported by reports in vascular smooth muscle cells where S-nitrosoglutathione (GSNO) exposure provokes nitrosation of residues within the 20S catalytic core of the 26S proteasome, known to contain 10 cysteines, which subsequently results in the inhibition of all three catalytic activities of the complex (chymotrypsin-, trypsin-, and caspase-like)^29^. Consistent with this, we observe S-nitrosation on cysteine residues encoded by proteasomal subunits within the 20S core as well as the 19S regulatory complex (Fig. 6B–D). Since cysteines are not directly involved in the catalytic mechanism of proteolysis, the effect of cysteine modification on enzymatic activity is most likely going to be indirect. NO-Cys-modifications have also been noted in the regulatory ATP (PSMD1, PSMC2, PSMC6) and non-ATP subunits (PSMD2, 6, 11, 12), suggesting potential alterations in substrate recognition and unfolding (Fig. 6). Modifications on regulatory subunits and core particle of the proteasome have been reported – General oxidation^36^ and Cys-glutathionylation interfere with proteasomal proteolysis^37^. Thioredoxin-related protein TNXL1 is part of the proteasome complex^38^ and may reverse unwanted Cys-modifications.

There are many reports that link the ubiquitin-proteasome system with the regulation of cardiovascular function, in both the heart and vessels. Cardiac hypertrophy, characterized by cell enlargement rather than proliferation, exhibits increased protein degradation by the ubiquitin-proteasome system, and dysfunctional proteasome function has been observed in heart failure, evidenced by the accumulation of ubiquitin-positive aggregates in failing hearts^39^ as well as levels of PA28 regulatory components^40^ Also, S-nitrosation of protein substrates can trigger degradation via the ubiquitin proteasome system, as has been shown for CDK5^41^, PDE5^42^ and caspase-3^43^.

In summary, our global profiling approach revealed widespread BH4-dependent S-nitrosation across the proteome affecting prominent enzymes within the UPS. This opens up possibilities of targeting BH4-dependent redox signalling to intervene in cardiovascular disease as a viable therapeutic strategy.

## Electronic supplementary material

Supplemental Figure 1

Supplemental Westerns

Supplemental Dataset
