# Supplementary material for: Tetrahydrobiopterin modulates ubiquitin conjugation to UBC13/UBE2N and proteasome activity by S-nitrosation
Source: Sci Rep. 2018 Sep 25;8:14310. doi: 10.1038/s41598-018-32481-4 (PMC6156325; doi:10.1038/s41598-018-32481-4)

Supplementary figure 1

Tetrahydrobiopterin modulates ubiquitin conjugation to UBC13/UBE2N and proteasome activity by S-nitrosation

Jade Bailey[a], Simon Davis[b], Andrew Shaw[a], Marina Diotallevi[a], Roman Fischer[b], Matthew A. Benson[a], Hanneng Zhu[a], James Brown[a], Shoumo Bhattacharya[a], Benedikt M. Kessler[b], Keith M. Channon[a] and Mark J. Crabtree[a]

[a]BHF Centre of Research Excellence, Division of Cardiovascular Medicine, Radcliffe Department of Medicine, John Radcliffe Hospital, University of Oxford, Oxford, OX3 9DU [b]Target Discovery Institute, Nuffield Department of Medicine, University of Oxford, Roosevelt Drive, Oxford, OX3 7FZ

Corresponding Author:
Dr Mark J. Crabtree
BHF Centre of Research Excellence, Division of Cardiovascular Medicine, Radcliffe Department of Medicine, John Radcliffe Hospital,
University of Oxford, Oxford, OX3 9DU.
Tel: +44(0)1865 287662
Email: mark.crabtree@well.ox.ac.uk

Running title: BH4-mediated S-nitrosation affects the UPS

Supplemental Figure 1

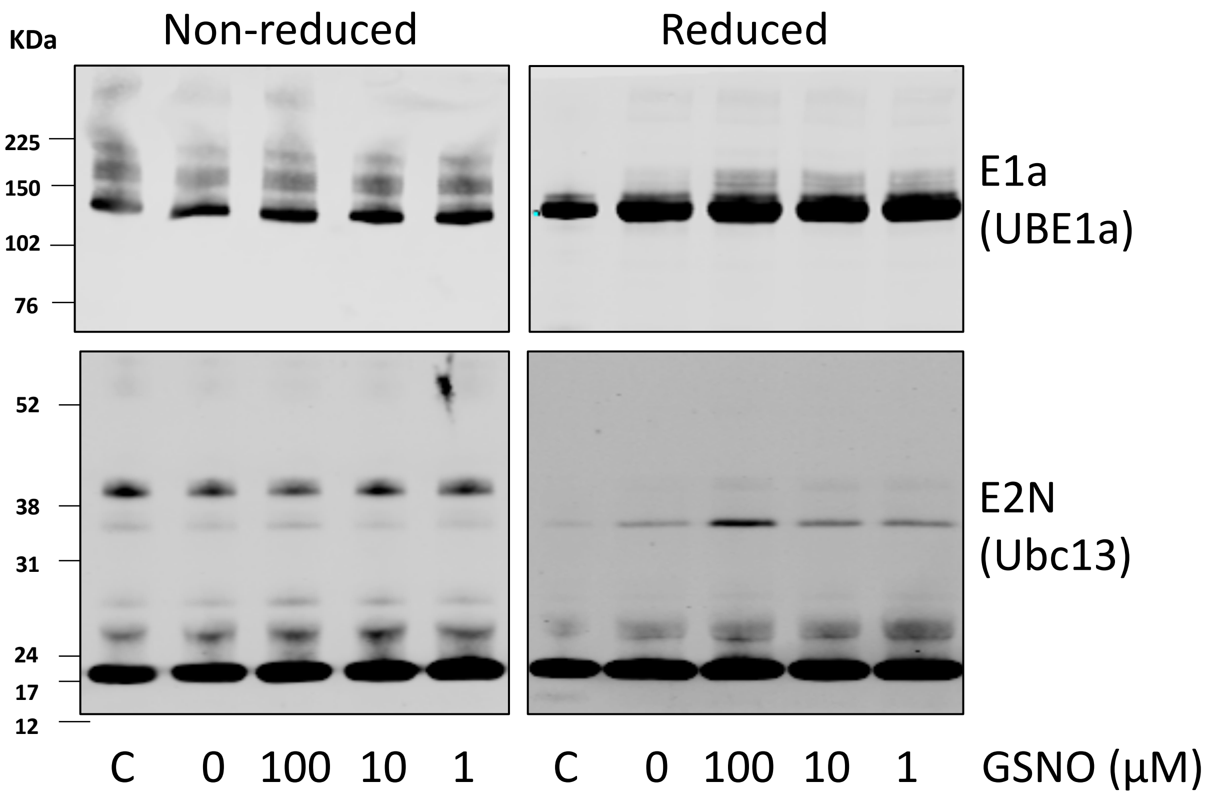

Supplement: Supplementary file 1 — Supplemental Figure 1 [file 41598_2018_32481_MOESM1_ESM.pdf]
